# Cannabinoid Receptor 2 Signaling Does Not Modulate Atherogenesis in Mice

**DOI:** 10.1371/journal.pone.0019405

**Published:** 2011-04-26

**Authors:** Florian Willecke, Katharina Zeschky, Alexandra Ortiz Rodriguez, Christian Colberg, Volker Auwärter, Stefan Kneisel, Melanie Hutter, Andrey Lozhkin, Natalie Hoppe, Dennis Wolf, Constantin von zur Mühlen, Martin Moser, Ingo Hilgendorf, Christoph Bode, Andreas Zirlik

**Affiliations:** 1 Department of Cardiology, University of Freiburg, Freiburg, Germany; 2 Forensic Toxicology, Institute of Forensic Medicine, University Medical Center Freiburg, Freiburg, Germany; Universität Würzburg, Germany

## Abstract

**Background:**

Strong evidence supports a protective role of the cannabinoid receptor 2 (CB_2_) in inflammation and atherosclerosis. However, direct proof of its involvement in lesion formation is lacking. Therefore, the present study aimed to characterize the role of the CB_2_ receptor in Murine atherogenesis.

**Methods and Findings:**

Low density lipoprotein receptor-deficient (LDLR^−/−^) mice subjected to intraperitoneal injections of the selective CB_2_ receptor agonist JWH-133 or vehicle three times per week consumed high cholesterol diet (HCD) for 16 weeks. Surprisingly, intimal lesion size did not differ between both groups in sections of the aortic roots and arches, suggesting that CB_2_ activation does not modulate atherogenesis in vivo. Plaque content of lipids, macrophages, smooth muscle cells, T cells, and collagen were also similar between both groups. Moreover, CB_2_^−/−^/LDLR^−/−^ mice developed lesions of similar size containing more macrophages and lipids but similar amounts of smooth muscle cells and collagen fibers compared with CB_2_^+/+^/LDLR^−/−^ controls. While JWH-133 treatment reduced intraperitoneal macrophage accumulation in thioglycollate-illicited peritonitis, neither genetic deficiency nor pharmacologic activation of the CB_2_ receptor altered inflammatory cytokine expression in vivo or inflammatory cell adhesion in the flow chamber in vitro.

**Conclusion:**

Our study demonstrates that both activation and deletion of the CB_2_ receptor do not relevantly modulate atherogenesis in mice. Our data do not challenge the multiple reports involving CB_2_ in other inflammatory processes. However, in the context of atherosclerosis, CB_2_ does not appear to be a suitable therapeutic target for reduction of the atherosclerotic plaque.

## Introduction

Atherosclerosis is a chronic inflammatory disease and represents the primary cause of heart disease and stroke worldwide [1]. While the inflammatory nature of atherosclerosis has been uncovered for sometime already, genuine anti-inflammatory treatment options are still lacking. Drugs with pleiotropic anti-inflammatory properties, such as statins, are cornerstones of current state-of-the-art therapy, while great efforts are made to find new agents primarily designed to abate the inflammatory and immunologic mechanisms promoting atherosclerosis and its complications. A growing body of evidence suggests that the cannabinoid system plays a critical role in the pathogenesis of inflammation and recent reports also implicated it with the pathobiology of atherosclerosis [2], [3], [4]. The endocannabinoid system comprises two membrane receptors, CB_1_ and CB_2_, their endogenous ligands, such as anandamide (arachidonoylethanolamide, AEA) and 2-arachidonoylglyceral (2-AG), and several enzymes required for their biosynthesis and inactivation [5], [6]. The receptor CB_1_ is primarily expressed in the central nervous systems (CNS), but also in peripheral tissues and on immune cells [7]. Selective blockade of the CB_1_ receptor inhibits atherogenesis in LDL receptor (LDLR)-deficient mice [8].

The CB_2_ receptor is predominantly expressed in immune and hematopoetic cells but also in adipose tissue [9], brain [10], myocardium [11], and endothelial cells [12]. Numerous studies have demonstrated anti-inflammatory effects of CB_2_ receptor activation in different diseases and pathological conditions, including cerebral injury [13], [14], inflammatory pain [15], and myocardial injury [16]. Most notably, CB_2_ receptor activation has also been suggested to modulate atherosclerosis [17]. In this latter study, Steffens and colleagues showed that oral administration of low doses of Δ^9^-tetrahydrocannabinol (THC, 1 mg/kg per day) significantly reduced plaque progression in apolipoprotein E (ApoE) knockout mice. They also observed CB_2_ receptor-expressing immune cells in Murine and human atherosclerotic plaques and reduced macrophage content in atherosclerotic lesions of THC-treated mice. Since these effects were reversed by a selective CB_2_ but not CB_1_ receptor antagonist, the authors hypothesized the involvement of CB_2_ receptors on immune cells in atherogenesis [17]. Another recent study also showed amelioration of atherosclerosis in ApoE-deficient mice after treatment with a CB_2_/CB_1_ receptor agonist and the authors postulated a CB_2_ receptor-dependent effect [3]. However, up to now no *in vivo* study evaluated the direct contribution of CB_2_ receptor signaling in the context of atherosclerosis. There is a need for such studies to ultimately evaluate whether CB_2_-targeted therapies may be suitable to fight atherosclerosis. Therefore, the aim of this study was to investigate the influence of the CB_2_ receptor on atherogenesis in low density lipoprotein receptor (LDLR)-deficient mice.

## Methods

### In vivo study

CB_2_-deficient mice and LDLR-deficient mice, both on a pure C57/BL6 background, were obtained from Jackson Laboratories. Mice were crossbred to generate CB_2_^−/−^/LDLR^−/−^ mice. Genotyping of each mouse used polymerase chain reaction and employed the following primers: LDLR, 5′-CCA TAT gCA TCC CCA gTC TT-3′ (common primer), 5′-gCg ATg gAT ACA CTC ACT gC-3′ (wild-type primer), 5′-AAT CCA TCT TgT TCA ATg gCC gAT C-3′ (mutant primer); CB_2_, 5′- gAC Tag AgC TTT gTA ggT Agg Cgg -3′ (common primer), 5′- ggA gTT CAA CCC CAT gAA ggA gTA-3′ (wild-type primer) and CB_2_, 5′- ggg gAT CgA TCC gTC CTg TAA gTC T-3′ (mutant primer). Six-week-old male CB_2_^+/+^/LDLR^−/−^ and CB_2_^−/−^/LDLR^−/−^ mice consumed a high-cholesterol diet (HCD) for 16 weeks (Ssniff modified after Research Diets D12108 containing 21% total fat and 1.235% cholesterol, Soest, Germany). In parallel, JWH-133 (diluted in a water-soluble solution with Tocrisolve, from Tocris, Bristol, UK) or vehicle was injected intra-peritoneally (i.p.) into LDLR-deficient mice consuming HCD at a concentration of 5 mg/kg body weight three times a week for 16 weeks. Subsequently, mice were euthanized, hearts and aortas were removed, and tissue was prepared and analyzed histologically as described previously [18], [19], [20]. Total cholesterol and triglyceride levels were assayed in EDTA plasma from blood obtained by retro-orbital bleeding before feeding and drawn from the right ventricle upon harvest. The total wall area ( = intima + media), intimal lesion area (intima) and medial area (media) as well as the percentage of positively stained area for macrophages (anti-mouse Mac-3), lipids (Oil-Red-O), T cells (anti-CD4), collagen (Picrosirius red), smooth muscle cells (anti-α-actin), and apoptotic cells (TUNEL, Roche, Basel, Switzerland) were quantified by blinded investigators employing computer-assisted image analysis software (Image Pro, Media Cybernetics, Bethesda, MD). Abdominal aortas were fixed with 10% formalin, opened longitudinally, pinned, stained with Oil-red-O solution (Sigma-Aldrich, St. Louis, MO, 2.5 h, RT), washed with 85% propylene glycol, and lipid accumulation was quantified as described above. All mice were housed under specific pathogen-free conditions. All procedures were approved by the Animal Care Committee of the University of Freiburg and the local authorities (Regierungspräsidium Freiburg) with the permit number G-08/46.

### Cell Culture and in vitro stimulation

Murine endothelial cells were isolated using Invitrogen Dynabeads® (Invitrogen, Paisley, UK) as previously described [21]. Endothelial cells were seeded in 6 or 24 wells until they reached 80% confluence. Flow cytometric analysis for PECAM-1 and ICAM-2 showed a purity of about 97% of isolated murine endothelial cells (data not shown). After incubation in FCS-free medium for 24 hours, cells were stimulated with indicated concentrations of JWH-133 or vehicle, followed by TNFα (20 ng/ml) after 30 minutes. After 24 hours of incubation at 37°C supernatents were removed for ELISA and endothelial cells were lysed and used for Western Blotting. Apoptosis and cytotoxicity was evaluated by Apo-ONE® and CytoTox-One™ Assay according to the instructions of the manufacturer (Promega, Madison, WI).

### Enzyme-linked immuno-absorbent assay (ELISA)

Mouse MCP-1 was quantified in the supernatants of cell cultures using commercially available ELISA Kits (R&D DuoSet, Minneapolis, MN) according to the manufacturer's instructions.

### Western blotting

Murine endothelial cells were stimulated with TNFα (20 ng/ml) for 24 hours. After incubation, murine endothelial cells were lysed, separated by SDS-PAGE under reducing conditions, and blotted to polyvinylidene difluoride membranes as described previously [21]. An anti-mouse ICAM-1 antibody (Santa Cruz, Santa Cruz, CA) was used as primary antibody, followed by an anti-peroxidase-conjugated AffiniPure Goat Anti-Rabbit IgG (Jackson Laboratories, West Grove, PA) as secondary antibody.

### Cytokine challenge and cytometric bead assay

To induce inflammation, mice were subjected to intraperitoneal injection of TNFα (200 ng/ml) as indicated. Blood was collected by cardiac puncture and serum separation. For analysis of inflammatory markers in mice the cytometric bead assay for Murine inflammation detecting IL-6, MCP-1, IFNγ, IL-10, and IL-12p70 was used according to manufacturer's instructions (BD Biosciences, San Diego, CA) optimized for higher sensitivity. Results were analyzed using the corresponding FCAP software (BD Franklin Lakes, NJ). The lower detection limits were in the range of 5–10 pg/ml.

### Dynamic adhesion assays

Dynamic adhesion assays in the flow chamber were performed as described previously [19]. Murine endothelial cells were grown in 35 mm dishes (Costar, Bethesda, MD) and were subjected to the flow chamber. In brief, the Glycotech flow chamber (Gaithersburg, MD) was assembled with the dish as the bottom of the resulting parallel flow chamber. The chamber and tubes were filled with PBS without serum prior to the experiment. Subsequently, Murine leukocytes were applied with a syringe pump (Harvard apparatus PHD2000, Holliston, MA) with flow rates of 0.04 dyne/cm^2^ (venous flow; a total of 10 min). Adherent cells were quantified under the microscope.

### Flow cytometry

Flow cytometry was performed as previously described [22]. Cells were pre-incubated with mouse Fc-Block (αCD16/32, ebioscience, San Diego, CA). Antibodies included CD11b-FITC, CD115-PE, Ly6C/G (Gr1)-APC, CD4-Alexa488, CD8-PE, CD3-APC, CD20-PE, ICAM-1-FITC (all from ebioscience, San Diego, CA), ICAM-2-FITC, and PECAM-1-PE (PharMingen, San Diego, CA). The mean fluorescence indices (MFI) were quantified employing the FlowJo software (Tree Star Inc, Ashland, OR).

### Murine peritonitis

CB_2_^+/+^/LDLR^−/−^ and CB_2_^−/−^/LDLR^−/−^ mice were treated intraperitoneally with 4% thioglycollate. After 4 and 72 hours, mice were euthanized with CO_2_, the peritoneal cavity was flushed with 6 ml of RPMI for 3 min. Leukocytes were quantified in a CASY counter. Similarly, wild-type (Bl6) mice received JWH-133 one hour before injection of 4% thioglycollate. The resulting leukocyte migration was measured after 4 and 72 hours.

### Mass spectrometry - pharmacokinetics of JWH-133

Mice were subjected to i.p. injection of JWH-133 (5 mg/kg body weight) on day 1, 3, 6, 8 and 10. On day 10 retro-orbital blood was taken at 2, 12, 24, and 48 hours after the last injection of JHW-133. Mouse sera (100 µl, N = 6) were added to 5 µl internal standard (d_3_-THC; 5 µg/ml in ethanol), followed by 2.9 ml acetic acid 0.1 M, followed by automated solid phase extraction using Aspec GX-274 (Gilson, Middleton, USA), reversed phase C_18_ SPE-cartouche (type chromabond), and a 500 mg column bed (Macherey-Nagel, Düren, Germany). Samples were eluted with 1 ml acetonitrile, evaporated in a stream of nitrogen at 40°C, and incorporated in 25 µl ethyl acetate.

Frozen aortic tissue was incubated in 500 µl ethanol in an ultrasound bath twice for 15 min, pooled, 25 ng internal standard was added (d_3_-THC), samples were evaporated in a stream of nitrogen at 40°C, reconstituted in ethanol, acetic acid was added, and automated solid phase extraction and elutriation was performed as described above.

1 µl of samples was analyzed using the Agilent 5973 GC-MS system (temperature gradient 100°C for 1 min, increase to 290°C for 2.5 min, increase to 310°C for 4 min, fragmentation energy 70 eV). The following fragments were chosen. For JWH-133: m/z 269 (quantifier), m/z 312 and m/z 229 (qualifier), retention time 6.5 min. For d_3_-THC: m/z 302 (quantifier), m/z 317 and m/z 234 (qualifier), retention time 7.4 min. The detection limit was 25 ng/ml.

#### Quantitative real-time PCR

Aortas from CB_2_^−/−^/LDLR^−/−^ and CB_2_^+/+^/LDLR^−/−^ mice consuming HCD for 16 weeks were harvested and stored in RNAlater (Qiagen, Venlo, Netherlands) at −80°C. Total RNA was extracted using TRIzol Reagent (Invitrogen, San Diego, CA) and glycogen as a co-precipitator (Roche, Basel, Switzerland). Homogenization was performed using a rotor-stator dispergator (IKA®, Staufen, Germany). 1 µg of total RNA was transcribed into cDNA using the Transcriptor First Strand cDNA Synthesis Kit (Roche, Basel, Switzerland). Subsequent quantitative real-time PCR was performed with a LightCycler 480 System using the LightCycler 480 SYBR Green I Master (Roche) detection format. mGAP-DH served as endogenous control. Primer sequences: mCNR1: 5′-TCC TTG TAG CAG AGA GCC AGC C-3′ (forward), 5′-GCC AGG CTC AAC GTG ACT GAG A-3′ (reverse); mGAP-DH: 5′-TGC ACC ACC AAC TGC TTA G-3′ (forward), 5′-GAT GCA GGG ATG ATG TTC-3′ (reverse).

### Statistical analysis

Data are expressed as means ± SEM of absolute or normalized values. Groups were compared employing the Student's t-test. A value of P<0.05 was considered significant. Data sets were analysed using GraphPad Prism® (GraphPad Software Inc, La Jolla, CA).

## Results

### Treatment with the selective CB_2_ receptor agonist JWH-133 does not attenuate atherogenesis in mice

**Figure 1:**
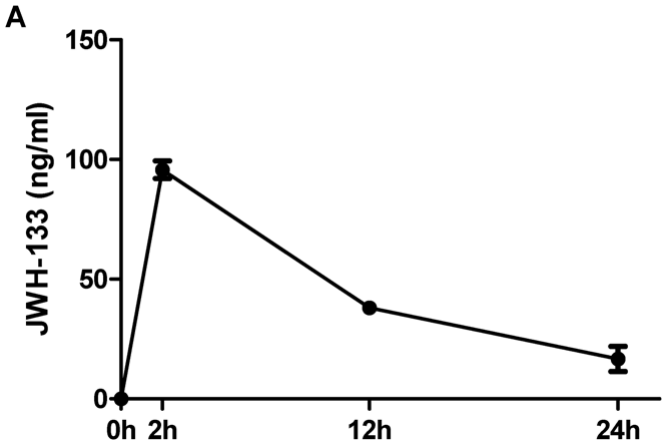
Pharmacokinetics of JWH-133 using mass spectrometry. Mice were subjected to intraperitoneal injection of JWH-133 (5 mg/kg body weight) on day 1, 3, 6, 8, and 10. On day ten, the serum levels of JWH-133 were determined at the indicated time points using mass spectrometry. The concentration of JWH-133 is given as the mean ± SEM (N = 6).

**Figure 2:**
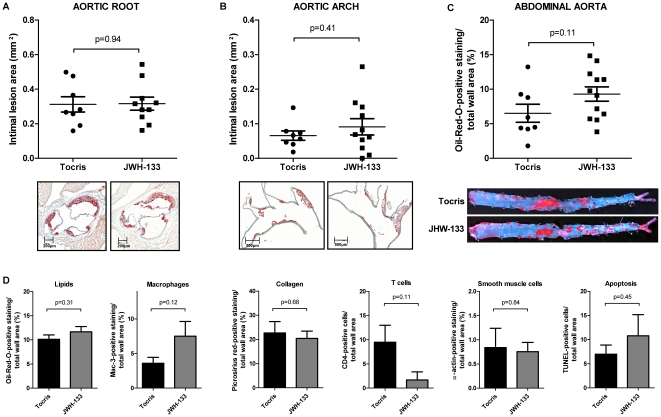
Treatment with the CB_2_ agonist JWH-133 does not modulate atherosclerosis in mice. A and B, LDLR^−/−^ mice consuming high cholesterol diet for 16 weeks (HCD) received intraperitoneal injections of 5 mg/kg JWH-133 (N = 10) or vehicle control (Tocris, N = 8) three times a week. Intimal lesion area in the aortic root (A) and arch (B) are diplayed as pooled data ± SEM; representative images stained for lipid deposition (Oil-red-O) are shown below the corresponding graph. C, The abdominal aortas of mice treated as described above underwent *en face* analysis of lipid deposition. Oil-red-O-positive staining in relation to total wall area was quantified and is displayed as pooled data ± SEM (N = 8 and 10); representative images are shown below. D, Sections of aortic roots of mice treated as described above were analyzed for lipid-, macrophage-, collagen-, T cell-, smooth muscle cell- and apoptotic cell content. Oil-red-O-, Mac-3-, picosirius red-, CD4-, α-actin- and TUNEL-positive staining in relation to total wall area is given as mean ± SEM (N = 8 and 10).

To explore the contribution of direct CB_2_ receptor stimulation LDLR^−/−^ mice consuming a high-cholesterol diet (HCD) for 16 weeks were treated with intraperitoneal injections of the selective CB_2_ receptor agonist JWH-133 or vehicle three times a week. JWH-133 was detectable in mouse serum after 2, 12, and 24 hours by mass spectrometry, proving bioavailability *in vivo* (Fig. 1). More importantly, JWH-133 could also be detected directly in aortic tissue of treated animals 48 hours after administration at a level of 2.2±0.67 ng/mg while it was undetectable in vehicle control-treated animals. Weights, cholesterol levels, and total leukocyte numbers did not differ between the study groups at baseline and end of feeding. Both groups also showed no difference in visceral fat mass, blood pressure, heart rate, and leukocyte subtypes as quantified at the end of the study (Table 1). Surprisingly, intimal lesion size in aortic roots was similar between JWH-133-treated mice and those receiving vehicle control (0.316±0.038 mm^2^, N = 10 vs. 0.312±0.044 mm^2^, N = 8, P = 0.94; Fig. 2A). Similar results were obtained in aortic arches (0.091±0.024 mm^2^, N = 11 vs. 0.066±0.013 mm^2^, N = 8, P = 0.41, Fig. 2B). Also, lipid deposition in *en face* analysis of abdominal aortas did not differ between both groups (Fig. 2C), demonstrating that CB_2_ receptor stimulation does not attenuate atherogenesis in mice. Similarly, JWH-133 treatment did not modulate the content of lipids, macrophages, collagen, T cells, smooth muscle cells, and the cellular apoptosis rates in atherosclerotic plaques (Fig. 2D).

**Table 1:** Characteristics of study animals before and after feeding.

		Tocris(N = 12)	JWH-133(N = 17)	p-value	CB_2_^+/+^/LDLR^−/−^ (N = 17)	CB_2_^−/−^/LDLR^−/−^(N = 18)	p-value
Weight (g)	BF	20.95±0.91	19.17±0.74	0.14	21.85±0.45	22.36±0.42	0.42
	AF	31.28±1.27	30.89±0.93	0.80	33.03±1.07	35.57±1.12	0.11
Cholesterol (mg/dl)	BF	191.5±11.82	201.0±11.82	0.59	194.7±11.47	183.2±7.66	0.41
	AF	907.6±120.9	1064.0±195.7	0.54	777.0±48.55	852.0±108.3	0.54
Triglycerides (mg/dl)	BF	98.84±11.67	146.0±15.78	0.04	163.9±21.29	137.1±9.23	0.26
	AF	255.8±50.95	219.2±23.13	0.48	194.5±21.81	202.7±21.36	0.79
Visceral fat pads (g)	AF	1.2±0.21	1.01±0.16	0.68	1.31±0.17	1.53±0.20	0.41
Systolic Blood Pressure (mmHg)	AF	102.8±3.77	107.1±6.04	0.57	98.96±2.49	104.2±3.86	0.27
Heart rate (bpm)	AF	679.8±17.78	634.6±17.01	0.08	615.2±16.16	659.1±18.79	0.09
Leukocytes (×1000/µl)	BF	10.73±0.79	10.71±0.85	0.98	9.77±0.57	10.93±0.55	0.15
	AF	4.5±1.02	6.49±0.72	0.11	6.92±0.89	6.36±0.55	0.59
CD3+ (% leukocytes)	AF	26.45±3.46	21.73±2.71	0.29	19.47±2.40	20.13±1.85	0.83
CD4+ (% leukocytes)	AF	12.55±1.71	9.4±0.66	0.07	8.47±0.87	9.75±0.78	0.28
CD8+ (% leukocytes)	AF	9.36±1.05	7.53±0.58	0.12	7.13±0.62	7.68±0.59	0.52
CD20+ (% leukocytes)	AF	12.27±2.27	19.13±3.52	0.15	12.0±1.85	19.19±4.71	0.18

### CB_2_ receptor deficiency does not affect the development of atherosclerotic lesions in mice

**Figure 3:**
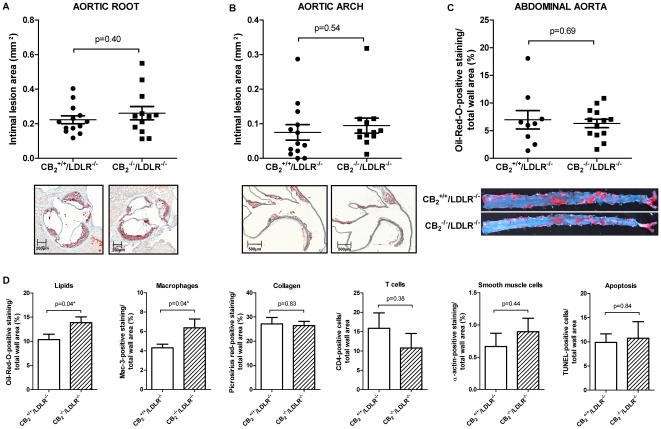
CB_2_ receptor deficiency does not influence atherosclerosis in mice. A and B, CB_2_^+/+^/LDLR^−/−^ (N = 13) and CB_2_^−/−^/LDLR^−/−^ (N = 12) mice consumed HCD for 16 weeks and underwent analysis of intimal lesion area in the aortic root (A) and arch (B). Pooled data ± SEM are shown on the left; representative images stained for lipid deposition (Oil-red-O) are displayed below the corresponding graph. C, The abdominal aortas of mice treated as described above underwent *en face* analysis of the lipid deposition. Oil-red-O-positive staining in relation to total wall area was quantified and is dispayed as pooled data ± SEM (N = 13 and 12); representative images are shown below. D, Sections of aortic roots of mice treated as described above were analyzed for lipid-, macrophage-, collagen-, T cell-, smooth muscle cell- and apoptotic cell content. Oil-red-O-, Mac-3-, picosirius red-, CD4-, α-actin- and TUNEL-positive staining in relation to total wall area is described as mean ± SEM (N = 13 and 12). Asterisks indicate a significant change, defined as p<0,05.

Consistent with our results for selective CB_2_ receptor stimulation, CB_2_^−/−^/LDLR^−/−^ mice consuming HCD for 16 weeks developed lesions of similar size as respective CB_2_^+/+^/LDLR^−/−^ control animals in aortic roots (0.261±0.038 mm^2^, N = 12 vs. 0.223±0.023 mm^2^, N = 13, P = 0.40, Figure 3A), aortic arches (0.095±0.022 mm^2^, N = 12 vs. 0.075±0.022 mm^2^, N = 13, P = 0.54, Figure 3B), and abdominal aortas (Fig. 3C). Again, there was no change in the degree of apoptosis, the content of collagen, T cells, and smooth muscle cells within the atherosclerotic plaque. However, we could detect increased lipid and macrophage content (Fig. 3D). CB_1_ expression quantified by RT-PCR did not differ between both groups rendering a CB_1_-driven bias unlikely (0.0013±0.0002 vs. 0.0018±0.0004, P = 0.34, N = 3). Study characteristics were similar between both groups at baseline and end of feeding (Table 1).

### CB_2_ receptor signaling differentially affects inflammatory cell recruitment

**Figure 4:**
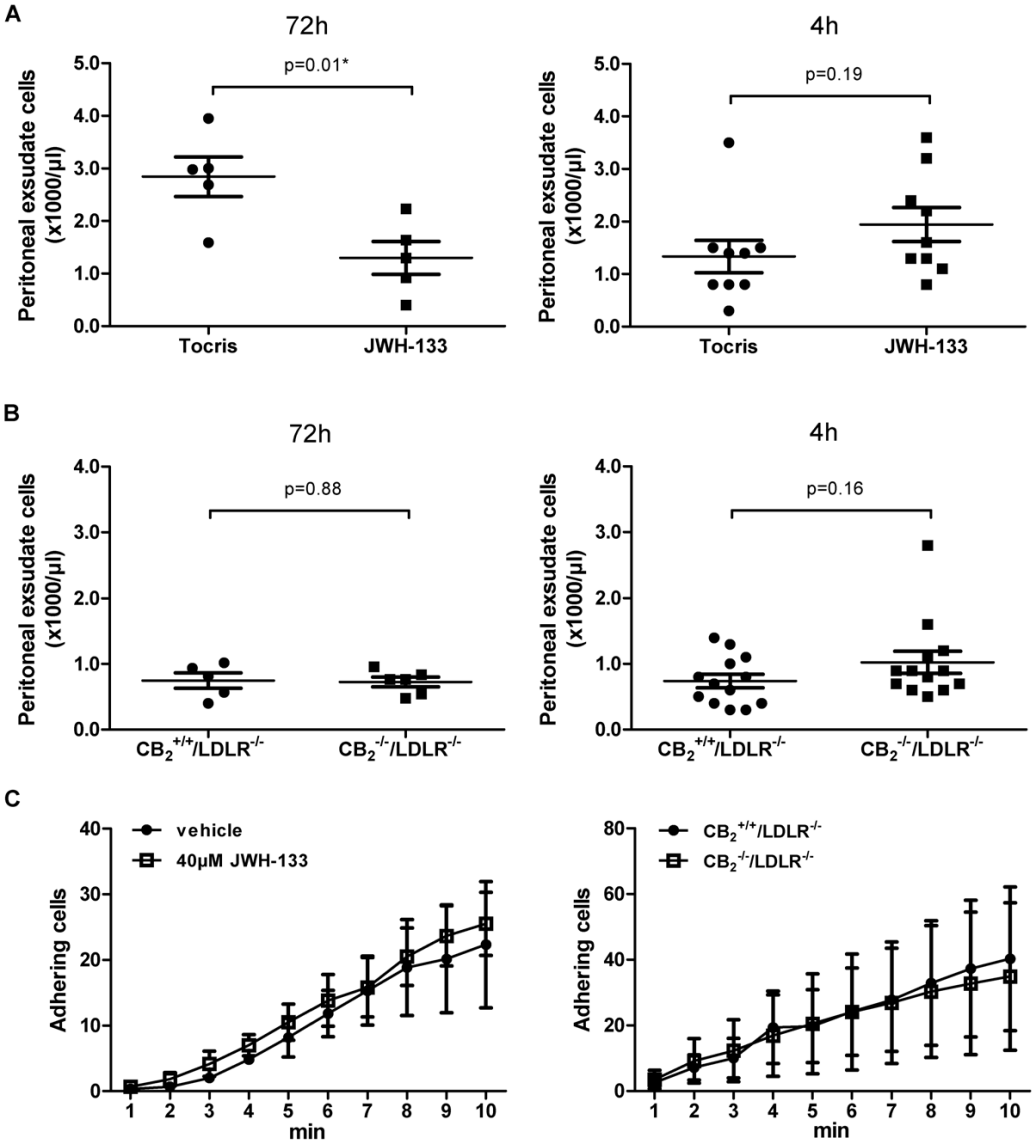
Inflammatory cell recruitment is differentially affected by CB_2_ receptor stimulation. A, Wild-type mice received intraperitoneal injections of 4% thioglycollate after pre-treatment with JWH-133 or vehicle control. Leukocyte recruitment into the peritoneal cavity was quantified after 72 and 4 h. Data represent mean ± SEM. Asterisks indicate significant change, defined as p<0,05. B, In parallel, thioglycollate-elicited accumulation of leukocytes in the peritoneal cavity was quantified in CB_2_^−/−^/LDLR^−/−^ mice and CB_2_^+/+^/LDLR^−/−^ control animals. Data for both 72 and 4 h stimulation are expressed as mean ± SEM. C, PMA-activated thioglycollate-elicited peritoneal leukocytes obtained from wild-type (Bl6) mice were allowed to adhere on TNFα-activated endothelial cells (EC) isolated by magnetic bead separation from wild-type mice in the presence or absence of 40 µM JWH-133. Adhering leukocytes were quantified under microscope after the indicated time points in the flow chamber (N = 3 each). In parallel experiments PMA-activated thioglycollate-elicited peritoneal leukocytes from CB_2_^−/−^/LDLR^−/−^ mice were allowed to adhere on TNFα-activated EC isolated from CB_2_^−/−^/LDLR^−/−^ mice. Adhesion was quantified and compared with the interaction of peritoneal leukocytes and EC isolated from CB_2_^+/+^/LDLR^−/−^ (N = 5 each). Pooled data represent mean ± SEM.

**Figure 5:**
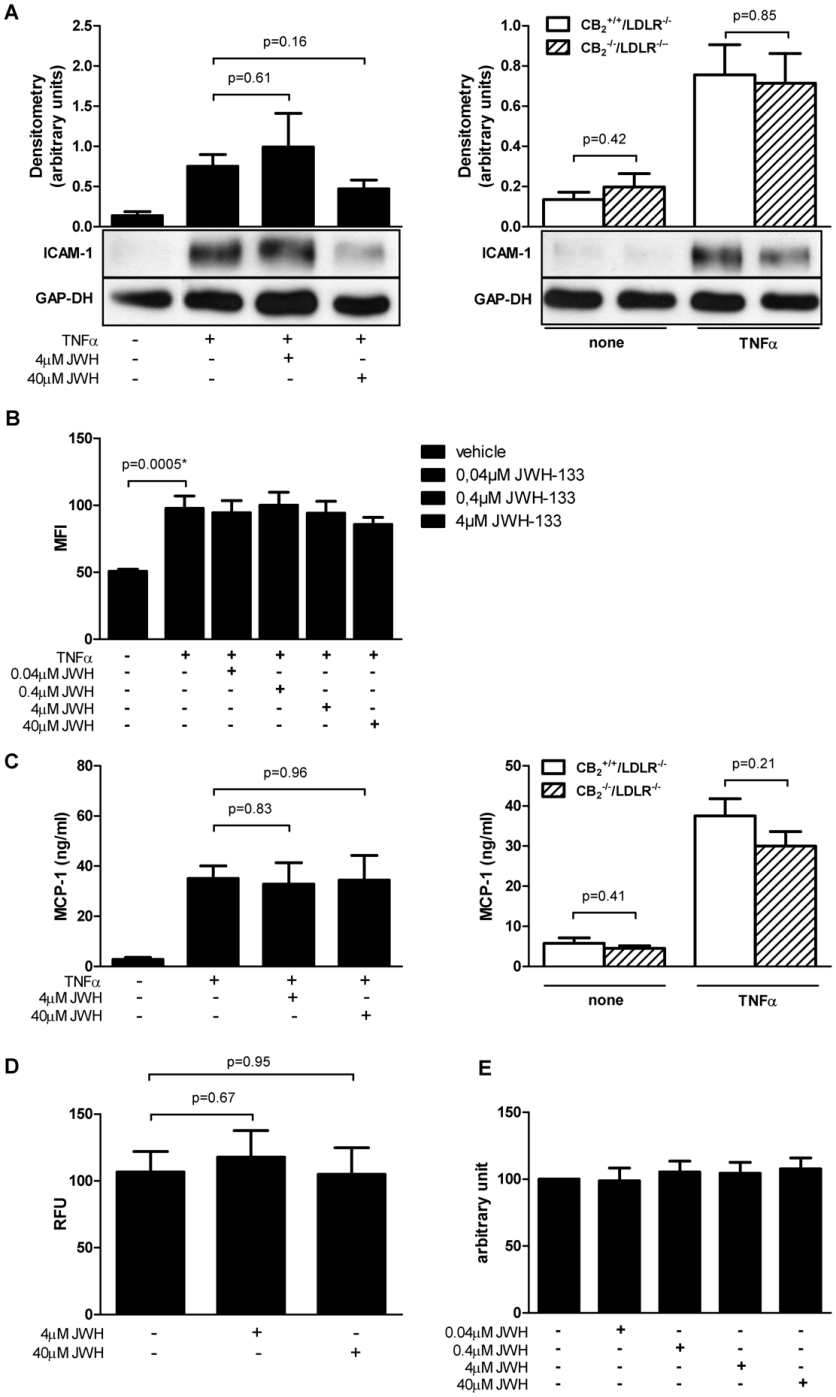
Viability and ICAM-1 expression on murine endothelial cells is unaffected by CB_2_ receptor signaling. A, Murine EC isolated from LDLR^−/−^ mice were stimulated with or without TNFα (20 ng/ml) and JWH-133 (4 µM and 40 µM, N = 4). In parallel, experiments, EC isolated from CB_2_^−/−^/LDLR^−/−^ mice and CB_2_^+/+^/LDLR^−/−^ control animals were stimulated with or without TNFα (20 ng/ml, N = 6). Cell lysates were analyzed for ICAM-1 by Western blotting. Western blots were analyzed densitometrically and adjusted for GAP-DH. Pooled data are given as mean ± SEM and representative blots are shown. B, Similarly, Murine EC isolated from wild-type mice where stimulated with indicated concentrations of JWH-133 and with or without TNFα (20 ng/ml). The cells were then analyzed for ICAM-1 expression using flow cytometric assays. Data is shown as mean ± SEM (N = 6). Asterisks indicate significant change, defined as p<0,05. C, In supernatants of EC treated as described above MCP-1 was quantified by ELISA. Data is shown as mean ± SEM. D and E, Murine EC isolated from wild-type mice were stimulated with indicated concentrations of JWH-133 and then the rate of apoptosis was determined using the Apo-ONE® Assay (D). Data is shown as the mean ± SEM (N = 5). The supernatant of cells treated in a similar manner were used to examine cytotoxicity with the CytoTox-ONE™ Assay (E). Data is shown as the percent of control (N = 6).

Since previous reports implicated the CB_2_ receptor in the recruitment of inflammatory cells, we investigated a potential role of CB_2_ receptor signaling in Murine peritonitis [12], [23], [24]. 72 hours after intraperitoneal injection of thioglycollate peritoneal macrophage numbers were significantly reduced in JWH-133-treated mice compared with vehicle controls (Fig. 4A N = 5 per group). In contrast, JWH-133 treatment did not affect short term (4 h) thioglycollate-induced peritonitis predominated by neutrophils (Fig. 4A, N = 9 per group). Similar amounts of leukocytes accumulated in the peritoneal cavity of CB_2_^−/−^/LDLR^−/−^ mice and CB_2_^+/+^/LDLR^−/−^ control animals after 72 and 4 hours (N = 5 and N = 13 per group, respectively, Fig. 4B). In accord, CB_2_ receptor signaling did not affect adhesion of inflammatory cells in the flow chamber (Fig. 4C), expression of ICAM-1 as assessed by Western blotting (Fig. 5A) and FACS (Fig. 5B), as well as chemokine expression in cultured endothelial cells (Fig. 5 C). JWH-133 did not modulate apoptosis (Fig. 5D) and cytotoxicity of the cells tested (Fig. 5E).

### Treatment with JWH-133 attenuates the recruitment of inflammatory monocytes to the blood pool in an acute Murine model of inflammation

**Figure 6:**
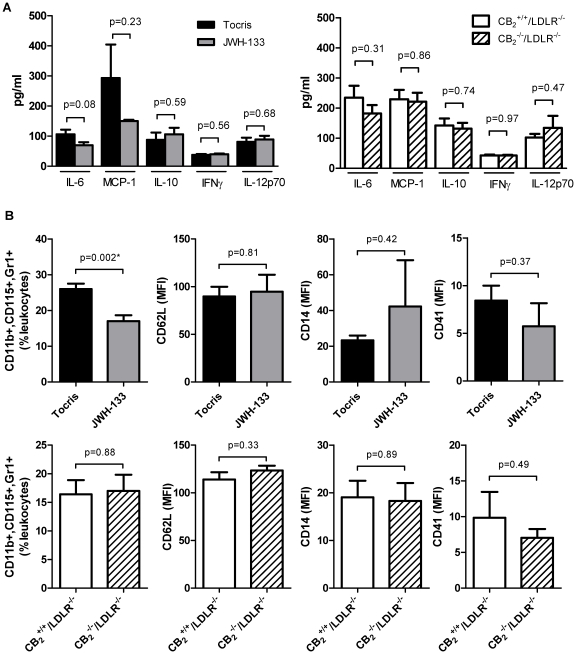
CB_2_ receptor stimulation or CB_2_ receptor deficiency does not affect inflammatory cytokine expression. A, LDLR^−/−^ mice treated intraperitoneally with 5 mg/kg JWH-133 or vehicle control (Tocris, N = 6 each) for 10 days were challenged with 200 ng TNFα for 4 hours. Subsequently, serum samples were analyzed for IL-6, MCP-1, IL-10, IFNγ, and IL-12p70 by cytometric bead array. In parallel, experiments the same analytes were quantified in CB_2_^−/−^/LDLR^−/−^ mice and CB_2_^+/+^/LDLR^−/−^ control animals after challenge with 200 ng TNFα for 4 hours (N = 5 each). Data represent mean ± SEM. B, Blood cells obtained from the animals treated as described above were quantified by FACS analysis for expression of CD11b, CD115, Gr-1, CD62L, CD18, CD14, and CD41. Data represent mean fluorescent intensity or per cent of leukocytes ± SEM where appropriate. Asterisks indicate significant change, defined as p<0,05.

Since we did not observe an effect of CB_2_ receptor signaling on atherosclerosis, a chronic inflammatory disease, we sought to explore its role in an acute model of inflammation. Interestingly, neither genetic deficiency nor selective stimulation of CB_2_ by JWH-133 modulated the expression of IL-6, MCP-1, IL-10, IFNγ, or IL-12p70 in mice challenged intraperitoneally with TNFα (Fig. 6A). However, JWH-133-treated mice recruited lower numbers of monocytes with an inflammatory, GR1^high^ subtype to the blood pool upon stimulation with TNFα. Of note, no difference in numbers of this cellular subtype could be measured between CB_2_^−/−^/LDLR^−/−^ and CB_2_^+/+^/LDLR^−/−^ mice.

## Discussion

The present study made the surprising finding that selective CB_2_ receptor stimulation did not affect the development of atherosclerotic plaques in LDLR-deficient mice. Accordingly, deletion of the CB_2_ receptor in LDLR-deficient mice did neither increase nor decrease atherosclerotic burden. There was a significant elevation of lipid and macrophage content in plaques of CB_2_^−/−^/LDLR^−/−^ mice, though. A recent study also showed no significant difference in atherosclerotic lesion area between CB_2_^+/+^/LDLR^−/−^ and CB_2_^−/−^/LDLR^−/−^ mice after 8 or 12 weeks on atherogenic diet. In accordance with our findings, plaques of these CB_2_-deficient animals contained more macrophages [25]. One possible explanation is that CB_2_ receptor deficiency reduces the susceptibility of macrophages to oxidzed LDL-induced apoptosis *in vitro* [26]. Therefore, the elevated macrophage levels in plaques of CB_2_^−/−^/LDLR^−/−^ mice might be the result of reduced apoptosis. Indeed, Netherland *et al.* observed decreased cellular apoptosis rates in atherosclerotic plaques from CB_2_^−/−^/LDLR^−/−^ mice [25]. In contrast, we could not detect CB_2_-dependent changes in apoptosis in our study animals. Increased macrophage content is a feature associated with more unstable plaques in humans. However, plaque stability also depends on collagen and smooth muscle content, which were both not modulated in our study. Furthermore, if CB_2_ deficiency results in more plaque inflammation and less stability, one would expect that CB_2_ agonism promotes less inflamed, more stable lesions. We could not observe such an effect in animals treated with JWH-133. In accord, i.p. application of the direct CB_2_ antagonist SR144528 in HCD-consuming ApoE^−/−^ mice did not modulate atherogenesis in another report [27]. Thus, while we cannot rule out that CB_2_ signaling may affect macrophage biology, in the context of atherosclerosis this does not appear to be relevant. Since CB_1_ receptor signaling is thought to be proatherogenic [8], [28], [29], we also quantified CB_1_ mRNA expression via RT-PCR, showing no significant difference between the CB_2_^−/−^/LDLR^−/−^ and CB_2_^−/−^/LDLR^+/+^ mice. This makes a CB_1_-driven bias unlikely, however we cannot rule out a change of receptor activation due to receptor internalization.

Our data challenge two previous reports suggesting CB_2_-dependent anti-atherosclerotic properties of endocannabinoids [3], [17]. Both studies observed only indirect evidence for a CB_2_-dependent effect and lacked the use of highly selective CB_2_ agonists or genetic CB_2_ knock-out animals. They demonstrated attenuation of atherosclerotic lesion formation by the CB_1_/CB_2_ agonists tetrahydrocannabinol (THC) and WIN55212-2, effects partially reversed by treatment with the CB_2_ antagonists SR144528 and AM630. Some reports claim selectivity of WIN-55,212-2 for the CB_2_ receptor but the compound also has a relatively high affinity for the CB_1_ receptor [30]. In contrast, JWH-133, used in this study, is a potent and selective CB_2_ receptor agonist, with a Ki of 3.4 nM and a 200-fold higher affinity for CB_2_ over CB_1_ receptors [31]. Numerous studies used JWH-133 *in vivo* at concentrations ranging from 0.015–15 mg/kg [14], [32], [33], [34], [35]. In the present study, we administered JWH-133 three times a week by intraperitoneal injection for the complete duration of high cholesterol diet, e.g. 16 weeks. This regimen resulted in detectable serum and aortic concentrations of JWH-133 as assessed by mass spectrometry, demonstrating bioavailability.

Imbalance in the ratio of the T cell subgroups and inflammatory monocytes as well as in their effector cytokines can modulate atherogenesis and plaque composition in mice [36], [37]. Several studies have shown that THC regulates Th1/Th2 cytokine balance in activated human T cells [7], [38], [39]. The expression of IFNγ was dose-dependently reduced in splenocytes after THC stimulation in a report whereas only a modest, non-significant down-regulation of IL-10 and TGFβ was detected, leading the authors to the conclusion that THC induces a dose-dependant shift in the Th1/Th2 balance [17]. Cytokine levels were too low to be quantified in our atherosclerosis model. To investigate whether deficiency or stimulation of the CB_2_ receptor has an influence on cells and cytokines also known to be involved in atherosclerosis, we chose a cytokine challenge model of acute Murine inflammation. The present study found no difference in IL-6, MCP-1, IL-10, IFNγ, and IL-12p70 expression after intraperitoneal TNFα challenge in both JWH-133-pretreated and CB_2_^−/−^/LDLR^−/−^ mice compared with respective controls. Therefore, in contrast to the non-selective THC, selective CB_2_ stimulation or deletion of the CB_2_ receptor has no influence on the expression of these cytokines *in vivo*. However, mice treated with JWH-133 for 10 days recruited lower numbers of inflammatory Gr1^high^ monocytes to the blood pool after intraperitoneal TNFα challenge, suggesting that CB_2_ stimulation may indeed have short term anti-inflammatory effects.

The recruitment of inflammatory cells (e.g., monocytes and T lymphocytes) to the intima is an essential step in the development and progression of atherosclerosis [40]. Rolling, adhesion, and trans-endothelial migration of leukocytes are triggered by local production of chemokines, chemokine receptors, and adhesion molecules [41]. Several previous *in vitro* studies have investigated the role of CB_2_ receptor activation on baseline or stimulated inflammatory cell migration, with both increases and decreases of cell migration reported, depending on the endocannabinoid, synthetic agonist/antagonist, and cell type used [for review, see 42]. Intraperitoneal injection of HU-210 and WIN-55,212-2 reduced the influx of neutrophils into peritoneal cavity in mice in one report [43]. However, both substances are considered to be both CB_1_ and CB_2_ agonists [44], [45], [46]. Using the highly selective CB_2_ agonist JWH-133, we detected a significant decrease of macrophage accumulation in the peritoneum of JWH-133-treated mice 72 hours after thioglycollate injection, suggesting anti-inflammatory properties of this drug *in vivo* at the dosage employed. In contrast, JWH-133 did not affect peritoneal neutrophil accumulation 4 hours after thioglycollate exposure. Accordingly, exposure of isolated LDLR^−/−^ endothelial cells to increasing concentrations of JWH-133 followed by TNFα stimulation did not mitigate MCP-1 and ICAM-1 expression. We could also not detect any significant differences in the expression of MCP-1 in TNFα-stimulated endothelial cells, isolated from CB_2_^−/−^/LDLR^−/−^ mice compared to respective controls. In contrast, Rajesh *et al.* found a significant decrease of MCP-1 and ICAM-1 in TNFα-stimulated human coronary artery endothelial cells after incubation with JWH-133 [12]. This might be due to cell type specific differences and methodical differences.

There are several limitations of this study that need to be considered: 1. Despite detection of JWH-133 in mouse serum and aortas by mass spectrometry, we cannot rule out that the dose of JWH-133 applied was insufficient to adequately stimulate the CB_2_ receptor *in vivo*. This is, however, unlikely since several other studies applied doses in a similar range to mice *in vivo* and observed biological effects [32], [33], [34], [35], [47]. Also, even if the dosage was insufficient one would still expect the genetic deficient animals to show an opposite effect which they did not in our study. 2. It is possible that CB_2_ receptor signaling affects intial but not later stages of atherosclerosis as tested in this study. However, one may question the biological and therapeutic relevance of such effects if they do not hold up through the course of atherogenesis. Also, plaques in the aortic arch are generally regarded to be at an earlier stage of development whereas those in the aortic root are considered to be more advanced [18]. Since we did not observe any modulation of atherosclerosis at both sites a stage-dependent effect is unlikely.

In summary, the present study made the novel observation that neither CB_2_ receptor stimulation nor its genetic deficiency modulates atherogenesis. Therefore, therapies targeting the CB_2_ receptor may not be beneficial in reducing atherosclerotic burden.
